# Biocompatible Multifunctional E-Skins with Excellent Self-Healing Ability Enabled by Clean and Scalable Fabrication

**DOI:** 10.1007/s40820-021-00701-8

**Published:** 2021-09-22

**Authors:** Xiuzhu Lin, Fan Li, Yu Bing, Teng Fei, Sen Liu, Hongran Zhao, Tong Zhang

**Affiliations:** State Key Laboratory of Integrated Optoelectronics, College of Electronic Science and Engineering, Jilin University, Changchun, 130012 People’s Republic of China

**Keywords:** Self-healing electronics, Strain sensors, Environmental friendly method, Multifunctional e-skins, Scalability

## Abstract

Electronic skins (e-skins) with an excellent sensing performance have been widely developed over the last few decades. However, wearability, biocompatibility, environmental friendliness and scalability have become new limitations. Self-healing ability can improve the long-term robustness and reliability of e-skins. However, self-healing ability and integration are hardly balanced in classical structures of self-healable devices. Here, cellulose nanofiber/poly(vinyl alcohol) (CNF/PVA), a biocompatible moisture-inspired self-healable composite, was applied both as the binder in functional layers and the substrate. Various functional layers comprising particular carbon materials and CNF/PVA were patterned on the substrate. A planar structure was beneficial for integration, and the active self-healing ability of the functional layers endowed self-healed e-skins with a higher toughness. Water served as both the only solvent throughout the fabrication process and the trigger of the self-healing process, which avoids the pollution and bioincompatibility caused by the application of noxious additives. Our e-skins could achieve real-time monitoring of whole-body physiological signals and environmental temperature and humidity. Cross-interference between different external stimuli was suppressed through reasonable material selection and structural design. Combined with conventional electronics, data could be transmitted to a nearby smartphone for post-processing. This work provides a previously unexplored strategy for multifunctional e-skins with an excellent practicality.

**Supplementary Information:**

The online version contains supplementary material available at 10.1007/s40820-021-00701-8.

## Highlights

- A printable multifunctional electronic skin (e-skin) with an excellent self-healing ability was developed via a simple, clean and universally applicable method.
- Water served as both the only solvent throughout the fabrication process and the trigger of the self-healing process. Each e-skin component was biocompatible.
- Combined with conventional electronics, signals collected by the e-skin could be transmitted to smartphones via Bluetooth for post-processing.

**Supplementary Information:**

The online version contains supplementary material available at 10.1007/s40820-021-00701-8.

## Graphical abstract

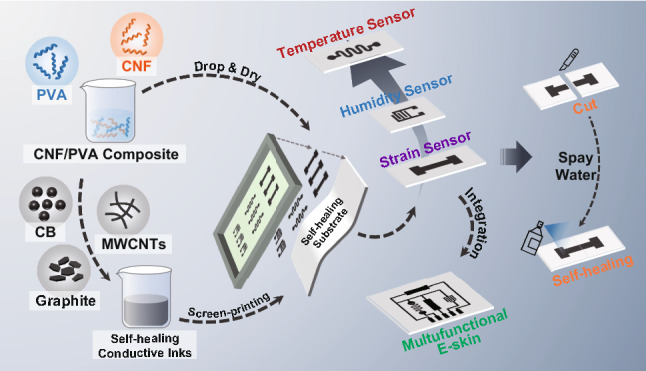

## Introduction

With the development of electronic technology, multifarious sensors have been integrated into our daily clothing and accessories, such as watches, glasses and bands, to facilitate effective and convenient real-time information collection. As one of the most ideal forms of future wearable electronic devices, electronic skins (e-skins), which exhibit an outstanding potential in the fields of health monitoring systems [1, 2], human–machine interaction [3, 4], artificial intelligence [5, 6] and robotics [7, 8], have drawn much attention in recent decades. When sensors are applied in e-skins, new demands occur. Similar to human skin, it is important for e-skins to sense external stimuli. E-skins with an excellent sensing performance have been widely developed over the last few decades. However, with the increasingly improved performance of sensors, practicality has become a new limitation [9–11].

In practical applications, friction, collision and excessive stretching of wearable electronic devices lead to mechanical damage causing degradation or even failure of the wearable electronic device. Self-healing ability endows e-skins with advantages such as a long-term robustness and reliability. However, most self-healable functional materials are incompatible with the traditional fabrication process [12]. Generally, bulk hydrogels of self-healable materials are directly applied as e-skins to achieve individual corresponding functions [13–16]. The main benefit of this approach is that the resultant e-skin can maintain a high self-healing efficiency so that the self-healed e-skin can almost maintain the original mechanical and functional properties. However, the poor processability of hydrogels results in difficulty when integrating various independent functional units. In other works, traditional functional materials have been deposited onto a self-healing polymer substrate via magnetron sputtering, evaporation, spray or drop casting [17–20]. Typically, the functional layer does not exhibit a self-healing ability, but the self-healing process of the substrate re-establishes contact between damaged functional layers to restore device function. Through this method, self-healable e-skins are compatible with the traditional fabrication process, which enables integration and the development of complex electronic circuits. However, the re-contacted part of the functional layer may break again upon bending or stretching to a certain degree. Bending and stretching almost inevitable occur in e-skins, especially in regard to strain sensing units responding to skin micro-deformation and monitoring bending or weak vibration signals produced by human movement and physiological activity [21–23]. Hence, e-skins must feature an autonomous repair capacity in practical applications [24]. Therefore, scalable and reliable fabrication processes such as patterning or printing must be developed to mass-produce self-healing devices and electronics. Considering that e-skins must tightly adhere to human skin and may be worn for a long time, they must be biocompatible. However, the use of certain additives and solvents in the fabrication of e-skin devices may result in bioincompatibility. Therefore, research on clean, environmentally friendly and scalable fabrication methods for biocompatible e-skins with an excellent self-healing ability should be explored.

Targeting the above problems, a multifunctional e-skin with a bilayer autonomous self-healing ability fabricated via the screen-printing technique was developed in this work. Various kinds of carbon materials, such as carbon black (CB), carbon nanotubes (CNTs) and graphite (G), were mixed with a self-healing binder to fabricate various functional units (e.g. electrodes, strain sensors and temperature sensors) on a self-healing substrate via the screen-printing technique. Considering that e-skins must adhere tightly to human skin and may be worn for a long time under all weather conditions, a composite of poly(vinyl alcohol) (PVA) and cellulose nanofiber (CNF), a biocompatible hydrogel with an excellent moisture-inspired self-healing ability, was applied both as the substrate and binder. Both the substrates and functional layers of e-skins are self-healable, which guarantees that self-healed e-skins can steadily operate even under a high strain. The screen-printing process of self-healable functional layers combines the advantages of two classical structures of self-healable devices. The planar structure is beneficial for integration and scalability, and the active self-healing ability of the functional layers endows self-healed e-skins with a higher toughness. Via the integration of different functional units onto one substrate, e-skins can achieve human motion monitoring and environmental temperature and humidity detection. Moreover, any cross-interference between different external stimuli can be suppressed through reasonable material selection and structural design of each functional unit. Water is the only solvent and trigger throughout the fabrication and self-healing process of e-skins. Our work presents a simple, universally applicable and pollution-free fabrication method for biocompatible e-skins with an excellent self-healing ability, integrating ability and scalability, which helps to promote more practical and environmentally friendly applications of e-skins in the human–machine interface and artificial intelligence fields.

## Experimental Section

### Materials

Poly(vinyl alcohol) (PVA-124) was purchased from Xilong Scientific Co., Ltd. Cellulose Nanofiber (CNF; 1.5 wt%; diameter: 5–20 nm; length: 1–3 μm) was acquired from ScienceK Co., Ltd. Nano-graphite powder (G; 99.9 wt%; thickness: < 40 nm; flow diameter: 3–6 μm) was obtained from XFNANO Technology Co. Ltd. Conductive CB (XC-72R; particle size: 30 nm) was purchased from Cabot Corporation. Multiwalled carbon nanotubes (MWCNTs; 95 wt%, outer diameter: 8–15 nm, length: ~ 50 μm) were acquired from Aladdin Industrial Inc. Deionized water was purified through a Millipore system.

### Fabrication of the CNF/PVA Self-Healing Substrate

Two millilitres of CNF aqueous suspension, 0.4 g PVA powder and 3.6 mL deionized water were mixed and stirred for 5 h at 90 °C. After the as-prepared CNF/PVA composite was cooled to room temperature, 3 g of the resultant CNF/PVA composite was dropped onto a polytetrafluoroethylene (PTFE) (25** × **75 mm^2^) mould and dried for 12 h at room temperature. The thickness of the CNF/PVA composite film is approximately 100 μm.

### Preparation of Self-Healing Electrodes

CB and G were chosen to prepare self-healing conductive ink for the electrodes. First, 0.2 g CB and 0.3 g G were added to a diluted CNF solution (2 mL CNF dispersion and 7 mL deionized water), and a uniform suspension was formed through stirring and ultrasonic treatment. Then, 0.4 g PVA was added to the suspension and stirred for 0.5 h at room temperature, followed by continuous stirring for 5 h at 90 °C. After the as-prepared CB/G-CNF/PVA composite was cooled to room temperature, it was patterned onto CNF/PVA self-healing substrates via screen-printing to prepare electrodes.

### Fabrication of Self-Healing Strain Sensors

Regarding the preparation of self-healing ink for strain sensors, G was chosen as the functional material. To investigate the influence of the G ratio on the sensitivity of the fabricated strain sensors, four kinds of inks based on G-CNF/PVA were prepared with different G concentrations. The G ratios were 50%, 60%, 70% and 80% (wt). The fabrication process of G-CNF/PVA ink is similar to the preparation process of CB/G-CNF/PVA ink. Strain sensors were also fabricated by screen-printing G-CNF/PVA ink onto CNF/PVA self-healing substrates.

### Fabrication of Self-Healing Temperature Sensors

MWCNTs were chosen as functional materials to fabricate temperature sensors. The fabrication process of MWCNTs-CNF/PVA ink is similar to the preparation process of CB/G-CNF/PVA ink. The final MWCNTs concentration in the solid composite was 50 wt%. A temperature sensor was fabricated by screen-printing MWCNTs-CNF/PVA ink onto a CNF/PVA self-healing substrate with a serpentine structure pattern.

### Fabrication of Self-Healing Humidity Sensors

First, interdigital electrodes were screen-printed onto a CNF/PVA self-healing substrate using CB/G-CNF/PVA ink. Then, the device was placed in an enclosed environment at 95% relative humidity (RH) for 30 min, after which a voltage of 4 V was applied between the two electrodes for 30 s with a DC voltage source. The resulting humidity sensor was dried at 11% RH after polarization.

### Characterization and Measurements

Fourier transform infrared (FT-IR) spectra of PVA and the CNF/PVA film were obtained on a WQF-510AFTIR spectrometer. Field emission scanning electron microscopy (FE-SEM) images were obtained with a JSM-6700F electron microscope (JEOL, Japan). Thermal gravimetric analysis (TGA) of the CNF/PVA composite was performed with a PerkinElmer thermal analysis system heated from 30 to 600 °C at a heating rate of 10 °C min^−1^ under an air atmosphere. A module slide and controller (EB1204 and CL-01A, respectively, HAIJIE Technology, Beijing) were employed to apply a bending stress to the strain sensors. Resistance and voltage signals were recorded with a digital measurement instrument (GDM-906X, GWINSTEK, Inc., Suzhou). A CHI660E electrochemical analyser (CH Instruments, Inc., Shanghai) was adopted to measure current–voltage (I–V) curves, and current–time measurements were achieved at a voltage of 1 V. The resistivity of the electrodes was measured with an ST2722-SZ four-probe tester (Suzhou Jingge Electronic Co., LTD).

## Results and Discussion

### Moisture-Inspired Self-Healing Performance of the CNF/PVA Composite

**Fig. 1:**
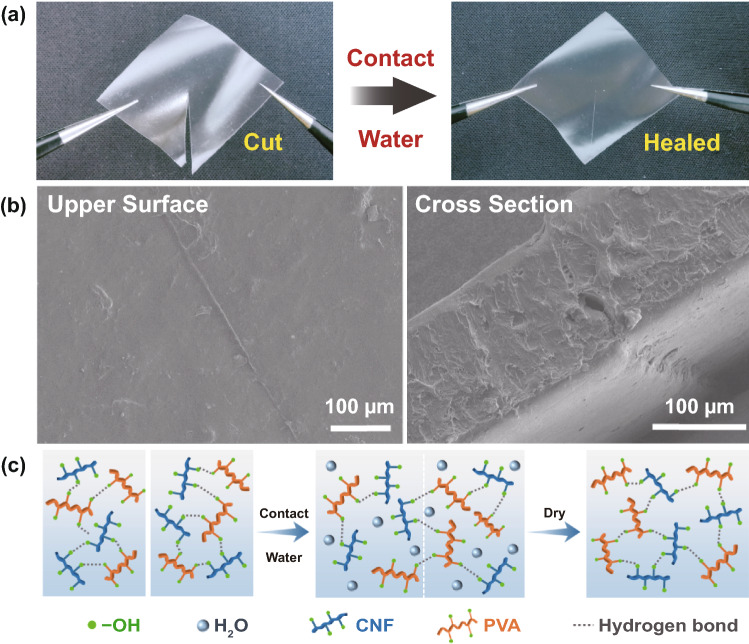
**a** Photographs of damaged CNF/PVA film (left) and self-healed CNF/PVA film (right). **b** SEM images of upper surface (left) and cross section (right) of self-healed position on CNF/PVA film. **c** Schematic illustration of the moisture-inspired self-healing mechanism of CNF/PVA film

The CNF/PVA composite was applied as both the substrate and binder of the functional layers in this work. Hence, the self-healing performance of CNF/PVA is crucial for self-healing e-skins. Photographs of damaged and healed CNF/PVA films are shown in Fig. 1a. The damaged CNF/PVA film self-healed within 10 min after the separated parts were re-connected and wetted with deionized water. To further observe the healed interface of the CNF/PVA film, SEM images of the healed section were obtained, as shown in Fig. 1b. Based on the images of the upper surface and cross section, the damaged section has completely healed. The mechanical strength of the healed material is one of the most important factors in evaluating the self-healing performance. Stress–strain curves of the CNF/PVA film (25** × **75 mm^2^, 100 μm in thickness) before and after self-healing were measured to investigate its mechanical strength (Fig. S1). Compared to the pristine CNF/PVA film, the strain at break of the healed CNF/PVA film slightly decreased. The healing efficiency of the CNF/PVA film was approximately 87%, indicating an excellent self-healing performance of the CNF/PVA composite. Here, the healing efficiency is defined as the ratio of the strain at break between the healed and original films. Moreover, stress–strain curve of the pure PVA film was measured. The strain at break and tensile modulus of the PVA film were lower than those of both the pristine and healed CNF/PVA films, indicating that the introduction of CNF not only endowed self-healing properties, but also improved the mechanical strength of the composite material.

The self-healing process and mechanism are shown in Fig. 1c. When the CNF/PVA film contacts water, the film rapidly swells via water absorption and transitions into the hydrogel state within a short time. However, at this moment, the CNF/PVA composite film has not yet healed, because the water molecules between the two surfaces hinder the formation of hydrogen bonds between the polymer molecules. After a few minutes, water is completely absorbed into the film, hydroxyl groups of the PVA and CNF chains are exposed at the surface, and a large number of hydrogen bonds are formed on the surface of the healing position [25]. The whole self-healing process is completed at room temperature (20 ℃, 15% RH). The chemical structure and thermal stability of CNF/PVA composite were also investigated by FT-IR (Fig. S2) and TGA (Fig. S3), and the details are shown in the Supporting Information. It should be noted that the thermal decomposition temperature of the composite is approximately 260 °C, which can meet the requirements of most practical applications.

To evaluate the self-healing ability of the CNT/PVA film, the self-healing efficiency of the CNT/PVA film and published self-healing materials are listed in Table S1. Via comparison, the self-healing efficiency of the CNF/PVA substrate is slightly lower than that mentioned in recent reports. In this work, to evaluate the self-healing efficiency of the obtained e-skins, a thin film, approximately 100 μm in thickness, was applied in self-healing efficiency measurements. We considered that the thin film sacrifices some of its self-healing efficiency because the contact area during the self-healing process is reduced. Although a small contact area is unfavourable during the self-healing process, the CNF/PVA film still exhibited a good self-healing efficiency of 87% after complete detachment, which indicates the good self-healing ability of our e-skins.

### Conductivity and Self-Healing Properties of the Electrodes

**Fig. 2:**
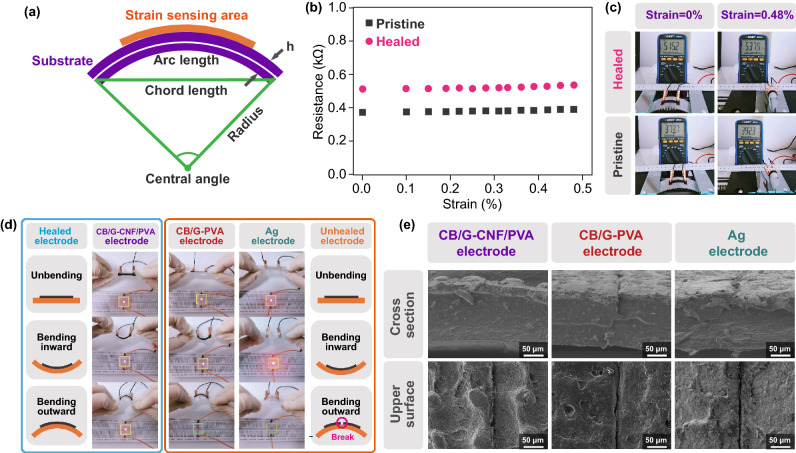
**a** Schematic illustration of calculation model of strain. **b** Resistance of pristine and self-healed electrodes under various strains and **c** under strain of 0% and 0.48% measured by a digital multimeter. **d** Visual controlling by using CB/G-CNF/PVA electrodes, CB/G-PVA electrodes and commercial silver electrodes. The different brightnesses of LEDs indicate bending states. **e** SEM images of self-healed position of CB/G-CNF/PVA electrodes, CB/G-PVA electrodes and silver electrodes.

An electrode, as an integral part of electronic devices, requires a good electrical conductivity. In regard to e-skins, the self-healing ability of electrodes directly determines the self-healing performance of the whole e-skin. Hence, higher requirements occur for electrodes. Here, CB and G were adopted as conductive materials, and CNF/PVA was applied as the binder to prepare screen-printable conductive ink. The electrical properties of the electrodes can be improved through the synergistic effect of hybrid conductive fillers since conductive fillers in different dimensions can increase the contact degree and interaction area between their interfaces [26, 27]. The detailed fabrication process is described in the Experimental Section. SEM images of the electrodes fabricated via the screen-printing technique on the CNF/PVA substrate are shown in Fig. S4. The region coated by the electrodes can be clearly distinguished from the blank substrate. As shown in the top view of the SEM image, the region coated by the electrodes (left side) is much rougher, and the smooth region (right side) indicates the surface of the CNF/PVA substrate. The SEM image of the cross section shows a uniform conductive layer fabricated via the screen-printing technique, which is much thicker than the substrate. The conductivity and self-healing performance of the electrodes were systematically investigated. The resistivity of the electrodes reached approximately 0.045 Ω cm. As shown in Fig. 2a, during the bending process, the strain value can be expressed as follows:

1 $$C = {2}R \times {\text{sin}}\left( {L/{2}R} \right)$$

2 $${\text{Strain}} = h/{2}R$$

where *C* and *L* denote the chord and arc lengths, respectively, of the device. The curvature radius (*R*) can be calculated with Eq. (1). Hence, the strain value of the device can be calculated with Eq. (2), where *h* is the thickness of the device. As shown in Fig. 2b, c, the resistance of pristine and self-healed electrodes was measured under various bending strains to investigate the self-healing performance and strain interference of the electrodes. The resistance of the pristine and self-healed electrodes remained almost unchanged even when the devices were bent to achieve an extremely large curvature. The maximum resistance drift of the pristine and self-healed electrodes was 5.0% and 4.3%, respectively, under a strain of 0.48%, which indicates that the electrodes are insensitive to strain.

In addition, the resistance of the electrodes increased after self-healing. The resistance of the electrodes was much lower than that of the other functional units in e-skins. Therefore, the slight resistance drift of the electrodes caused by the self-healing process did not affect the e-skin performance. To demonstrate the necessity of the self-healing ability of electrodes, the mechanical stability of self-healable electrodes and electrodes without a self-healing ability was investigated. Electrodes fabricated with CB/G-CNF/PVA ink, CB/G-PVA ink and commercial silver ink were cast onto CNF/PVA substrates via the screen-printing technique in the following measurements. Among these electrodes, the latter two electrodes are without self-healing ability. These three electrodes on substrates were cut into two pieces. Then, the electrodes were healed with substrates by spraying water. As shown in Fig. 2d, the three healed electrodes were inserted into a circuit containing a light-emitting diode (LED), and the conductivity of the electrodes was assessed based on the LED brightness. The schematic diagram of the equivalent circuit is shown in Fig. S5. (1) When the electrodes were unbent, the three LEDs were activated, and the conductivity of the three electrodes was restored. In this case, the damaged parts of the electrodes were re-contacted by the self-healing ability of the substrates regardless of whether the electrodes had completely healed, so the electrodes remained conductive. (2) When the electrodes were bent inwards, the conductivity of the three electrodes was unaffected, and the damaged parts of the conductive layers were compressed inwards. Hence, the electrodes still maintained a good conductivity. (3) When the electrodes were bent outwards, only the LED connected to the CB/G-CNF/PVA self-healing electrodes remained activated, while the LEDs connected to the electrodes without a self-healing ability were deactivated and the circuits were opened, which indicates that the electrodes broke again at the damaged location during outward bending. SEM images of the three types of healed conductive layers are shown in Fig. 2e. Based on the images of the cross section, it is observed that the substrates had all completely healed. The healing region of the electrodes was also observed in top and cross-sectional views. A healing trace was noted for the CB/G-CNF/PVA electrodes, but there was no crack in the healed region. Through comparison, obvious cracks appeared in the healed region of the CB/G-PVA and silver electrodes.

### Strain Sensing Performance and Self-Healing Properties of the Strain Sensor

**Fig. 3 Please replace figure 3 with attachment file.**
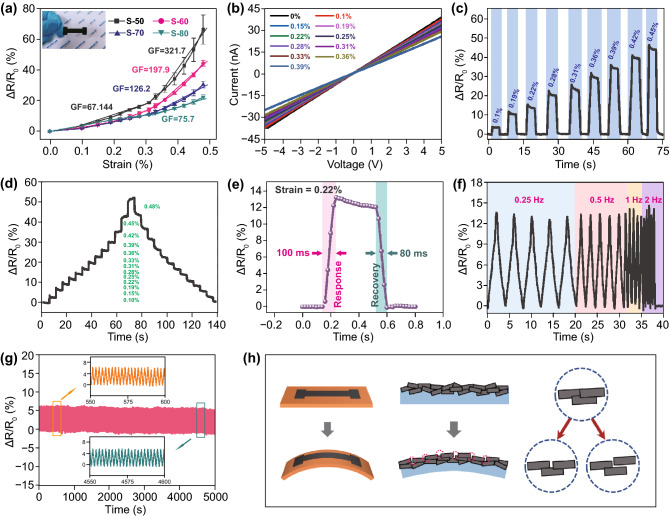
**a** Response vs strain curves of S-50/60/70/80 sensors. **b** I–V curves of the S-50 sensors under strains from 0 to 0.39%. **c** Dynamic response curves of S-50 sensor under various applied strains. **d** Dynamic response curve under a stair-like strain change. **e** Fast response and recovery speed of S-50 sensor. **f** Relative resistance variations under cyclic loading–unloading with a strain of 0.19% at the frequency of 0.25 to 2 Hz. **g** Current–time curves of the S-50 sensor for 5000 loading/unloading cycles with an applied strain of 0.25%. **h** Schematic diagram of the strain sensing mechanism

The strain sensor is a vital part of e-skins, which can respond to micro-deformation and can be applied in bending or weak vibration signal monitoring. Self-healable strain sensors based on the CNF/PVA substrate and G-CNF/PVA conductive ink were fabricated to investigate the sensing performance and self-healing properties of the strain sensing unit of e-skins. A photograph of the obtained strain sensor is shown in the inset of Fig. 3a. Strain sensors with different G concentrations (50%, 60%, 70% and 80%) were fabricated and denoted as S-50/60/70/80 to explore the influence of the G concentration on the strain sensing properties. In theory, strain sensors based on carbon nanomaterial-filled hydrogels can exhibit the maximum sensitivity at the percolation threshold concentration [28, 29]. However, in this work, to maintain the resistance of the strain sensors within an appropriate range, G concentrations from 50 to 80% were selected during fabrication. In regard to the strain sensors fabricated with the different inks, response versus strain curves of the four kinds of sensors are shown in Fig. 3a. The response is defined as *ΔR/R*_*0*_ (%) = *(R–R*_*0*_*)/R*_0_*100%, where *R*_*0*_ is the resistance of the sensor in the initial state and *R* is the resistance in the bending state at the various curvatures. All the curves demonstrate that the response of the sensors increased with increasing strain. To evaluate the sensitivity of the strain sensors, the gauge factor (GF) was calculated, which is defined as *GF* = *ΔR/(R*_*0*_*Δε)*, where *ΔR* and *Δε* denote the change in the resistance and strain, respectively. The sensitivity of the sensors decreased with increasing G concentration. Among the sensors, the S-50 sensors attained the highest GF within the whole strain range, the GF in the 0.31% ~ 0.48% strain range was 321.7, and the GF was 67.1 under a strain lower than 0.31%. When the G concentration was below 50%, the resistance of the strain sensors rapidly increased to an extremely high level with decreasing G concentration, which is unfavourable for measurements and leads to a high consumption in applications. Hence, the S-50 sensors were chosen as the optimal device in the following measurements.

I–V curves of the S-50 sensors under strains ranging from 0% to 0.39% were measured, as shown in Fig. 3b (voltage: ‒5 ~ 5 V). It can be observed that the I–V curves under the different strains exhibit a good linearity and ohmic behaviour. The slope of the I–V curves decreased with increasing strain, suggesting an increase in resistance. Dynamic response curves of the S-50 sensor under the various applied strains were measured, as shown in Figs. 3c and S6. The sensor exhibited an obvious response under the different strains. The response value increases with increasing strain, which is in accordance with the result shown in Fig. 3b. As shown in Fig. S6, the dynamic response and recovery curves of the S-50 sensor reached nearly the same response value in five measurement cycles under each applied strain, indicating a good repeatability of the sensor. The dynamic response curve under a stair-like strain change is shown in Fig. 3d. The response curve exhibits a stair-like rise and fall with the strain. In the bending and recovery process, the response values under the same strain are coincident, which demonstrates the excellent response and recovery properties of the S-50 sensors. The response and recovery speed is one of the most important parameters for strain units of e-skins applied in human health monitoring because the frequency of certain physiological signals, such as pulse, heartbeat and respiration, ranges from 0.2 to 2.0 Hz. Figure 3e shows a single response and recovery process of the S-50 sensor under a strain of 0.22%, and the response and recovery times are approximately 100 and 80 ms, respectively. Moreover, the effect of the strain rate on the sensing performance of the S-50 sensors was assessed, as shown in Fig. 3f. The response value of the S-50 sensors to a strain of 0.19% at the different strain rates remained almost invariant, so the sensor response was unaffected by the strain rate at frequencies ranging from 0.25 to 2.00 Hz. As shown in Fig. S7, the S-50 sensors can respond to an extremely low strain, and the resistance of the sensors exhibits an obvious change when a strain of 0.03% is applied. The mechanical durability is another crucial performance factor of strain sensors, especially in practical applications. As shown in Fig. 3g, the durability of the S-50 sensors was tested by repeatedly loading–unloading a strain of 0.25% over 5000 cycles, and the sensor maintained its sensing performance throughout 5000 cycles of the loading–unloading process. Based on the enlarged curves in insets, there is no notable difference between the anterior 50 s and posterior 50 s periods of the whole measurement curve, indicating the outstanding durability and reliability of the S-50 sensors. Considering the stretching or compressing caused by strain may occur at local stress concentration positions in strain sensors, which can affect the sensor thickness and cause system errors. The thickness distribution of the CNF/PVA film under bending was also evaluated (Fig. S8). The system error caused by the thickness variation during the strain-sensitive unit performance measurement is calculated far less than 1.39% (Details are discussed in Fig. S8).

A schematic diagram of the strain sensing mechanism is shown in Fig. 3h. When a bending strain is applied to the sensor, relative sliding may occur between the graphite sheets of the sensitive layer. If the strain reaches a high level, the contact area between the G sheets decreases, which enhances the resistance change in the strain sensors. When the G concentration of the sensitive layer is high, the reduction in contact area does not greatly influence the resistance of the strain-sensitive layers. With decreasing G concentration, strain can cause more damage on the conductive channel in the sensitive layer, resulting in the higher response of sensors in lower G concentration.

**Fig. 4:**
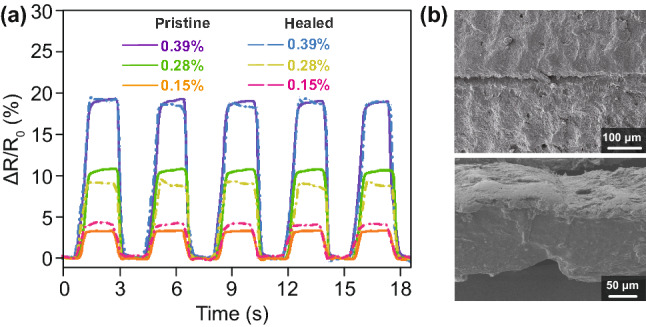
**a** Dynamic response curves of pristine and self-healed S-50 sensors under different applied strains. **b** Vertical (top) and cross-sectional (bottom) SEM images of S-50 sensors

It is a challenge for strain sensors to maintain their strain sensing performance and good mechanical stability after self-healing, because strain sensors always operate under various strain degrees. Hence, the self-healing ability of the S-50 sensors was studied. As shown in Fig. 4, the strain sensing performance of pristine and self-healed sensors was compared, and dynamic response curves measured under the different applied strains are shown in Fig. 4a. The self-healed sensor exhibited a response trend similar to that in its pristine state, and the maximum drift of the response value was 16.3%. In addition, SEM images of the self-healed sensor are shown in Fig. 4b. Both the strain-sensitive layer and substrate were well repaired after the self-healing process. The above results illustrate that the mechanical and sensing characteristics of the sensor were recovered after self-healing. Considering the interference of temperature on the strain sensing performance of sensors in practical applications, especially for multifunctional e-skins, response–strain curves of the S-50 sensors were measured at various temperatures, as shown in Fig. S9. Within the temperature range from 20 to 50 °C, the S-50 sensors did not exhibit an obvious variation in the strain sensing performance.

**Fig. 5 Please replace figure 5 with attachment file.**
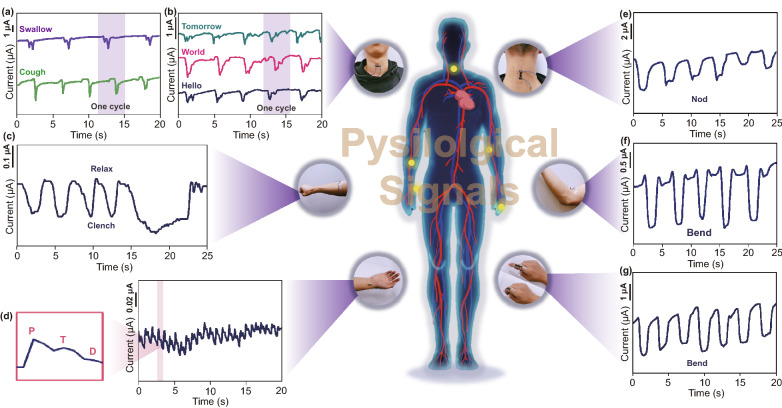
**a** Current response to the swallow and cough by attaching the strain sensors on the throat. **b** Voice and speech recognition by attaching the strain sensors on throat near the vocal cord. **c** Detection of holding–loosing the fist by attaching the strain sensors on the arm. **d** Real-time monitoring of the radial artery pulse by attaching the strain sensors to the wrist. The enlarged image (left) is single cycle of radial artery pulse wave form including “P”, “T” and “D” peaks. **e** Detection of nod by attaching the strain sensors to the posterior neck. **f** Detection of elbow bend by attaching the strain sensors to the joint of elbow. **g** Detection of finger bend by attaching the strain sensors to the knuckle

To a certain extent, physiological signals can reflect the health of the human body. Real-time monitoring of physiological signals is helpful to discover changes in physical function and plays an important role in medical diagnosis and disease prevention. Every human action leads to different degrees of skin stretching or retraction at corresponding positions, and the strain sensor can deform with the epidermis by being attached to the skin surface. Similarly, certain physiological signals producing weak vibrations can also be transmitted to the sensor through the skin. Hence, sensors with an excellent sensitivity are necessary to accurately detect physiological signals. To explore the potential of the self-healing strain sensor in wearable applications, the sensor was attached to skin at different positions to collect the various strain signals produced by the human body. As shown in Fig. 5a, the sensor was affixed to the neck of a tester to collect the weak signals caused by the deformation and vibration of muscles near the throat during the swallowing and coughing process. The thusly obtained swallowing and coughing curves reveal their own features. Moreover, vibration signals of the vocal cords can be detected by the S-50 sensors, which enables voice and speech recognition. As shown in Fig. 5b, different words such as “hello”, “world” and “tomorrow”, can be distinguished. Each word is repeated five times, and the characteristic waveforms of the same word are consistent, while the waveforms of the different words exhibit specific characteristic curves. In addition, as shown in Fig. 5c, the tiny muscle deformation produced from holding–loosing the fist was tested by attaching the sensor to the arm. Moreover, pulse signals can be collected by the S-50 sensors, and the curves are shown in Fig. 5d. The pulse of the radial artery on the wrist can directly reflect the heart rate, at the same time, in the enlarged view of a single-cycle pulse, the unique waveform of the pulse in each cycle contains three characteristic peaks, namely P (percussion wave), T (tidal wave) and D (diastolic wave) peaks, and the relationship among these three peaks is related to the blood pressure and certain heart functions [30, 31]. Apart from the detection of weak vibration signals, the sensor can be employed in the detection of large deformation, such as joint bending motion. The sensor has wide detection range, which is crucial for the strain sensor, to detect large-deformation signals. As shown in Fig. 5e–g, the sensor was attached to the posterior neck, elbow and fingers, real-time electrical signals of the strain sensor were recorded, obvious decreased current signals were produced by bent joints, and the current signals recovered when the joints were straightened. Therefore, the strain sensor exhibits a great potential application in the field of health monitoring and human–machine interaction. It should be noted that the strain sensor deformation degree during body movement monitoring and physiological signal detection is more rational than that during the standard strain sensing performance measurement process. The response of the strain sensors in Fig. 5 does not precisely reflect the strain value. However, we can still observe the relationship between physiological signals and the response of the strain sensors.

### Sensing Performances and Self-Healing Properties of the Temperature and Humidity Sensors

To expand the function of e-skins and verify that the fabrication technique of the above electrodes and strain sensing units is universally applicable to other functional units, temperature and humidity sensors were fabricated on CNF/PVA substrates. Here, the investigation focused on the self-healing properties of the functional units. Considering the interference attributed to the strain applied to the sensors, MWCNTs, a classical thermosensitive material, were chosen as the functional material for the temperature sensing layer. The 1D structure of MWCNTs can reduce the influence of the strain on the resistance of the temperature sensor. Furthermore, as shown in the inset of Fig. S10a, the sensitive layer of the temperature sensor based on MWCNTs-CNF/PVA is designed as a serpentine structure to further reduce the interference due to strain. The specific preparation process is described in the Experimental Section. The response of the temperature sensor within the temperature range from 20 to 45 °C is shown in Fig. S10a. The response of the temperature sensor is defined as *ΔI/I*_*0*_(%) = *(I − I*_*0*_*)/I*_0_*100%, where *I*_*0*_ is the current of the sensor at the initial temperature (20 °C) and *I* is the current at the various temperatures. The resistance of the temperature sensor decreased with increasing temperature because MWCNTs exhibit semiconductor properties, leading to a negative temperature resistance effect. The sensitivity of the temperature sensor was approximately 0.1%/°C, and the sensitivity is defined as *S* = *ΔI/(I*_*0*_*ΔT)*, where *ΔT* denotes the absolute temperature difference. Dynamic response curves of the temperature sensor in three heating and cooling cycles between 20 and 40 °C under the different strains were measured, as shown in Fig. S10b. The response values of the temperature sensor remained almost unchanged under the different bending degrees. It was thus verified that the temperature sensor was insensitive to bending deformation. Moreover, the self-healing ability of the temperature sensor was measured, as shown in Fig. S10c. The real-time response curves of the pristine and self-healed sensors almost coincided, indicating that the electrical and sensing characteristics of the temperature sensor had been perfectly recovered after self-healing.

Regarding the humidity sensor unit, a moisture-electric polarization process was applied to create a gradient of oxygen-containing groups on CNF/PVA film [32, 33]. The diffusion process of hydrogen ions driven by the concentration gradient can generate a potential difference between the electrodes. The potential difference is proportional to the water content of the polarized CNF/PVA film. The equilibrium water content of the CNF/PVA film is influenced by the RH. Hence, the RH can be measured with the polarized CNF/PVA film. The specific fabrication process of the humidity sensor is illustrated in Experimental section. As shown in Fig. S11a, the voltage increased with increasing RH, and the humidity sensor still exhibited a response to various RH after self-healing. Real-time voltage signals of pristine and healed sensors are shown in Fig. S11b, c, with the RH ranging from 75 to 95%. Considering that the effect of the temperature on the humidity sensor cannot be avoided in practice, voltage–RH curves at different temperatures were measured, as shown in Fig. S11d. The slope of the voltage–RH curves gradually increased with the temperature. Moreover, with increasing temperature, the humidity sensor responded to lower RH, and the voltage–RH curves tended to become saturated at high RH. All of the above phenomena are attributed to the absolute humidity increasing with the temperature at certain RH. Therefore, the humidity sensor can absorb more water molecules and exhibit a higher voltage to RH changes at higher temperatures. To achieve precise RH data, the temperature coefficient can be applied to calibrate the acquired electrical signal.

### Wearable and Self-Healing Multifunctional E-Skins

**Fig. 6:**
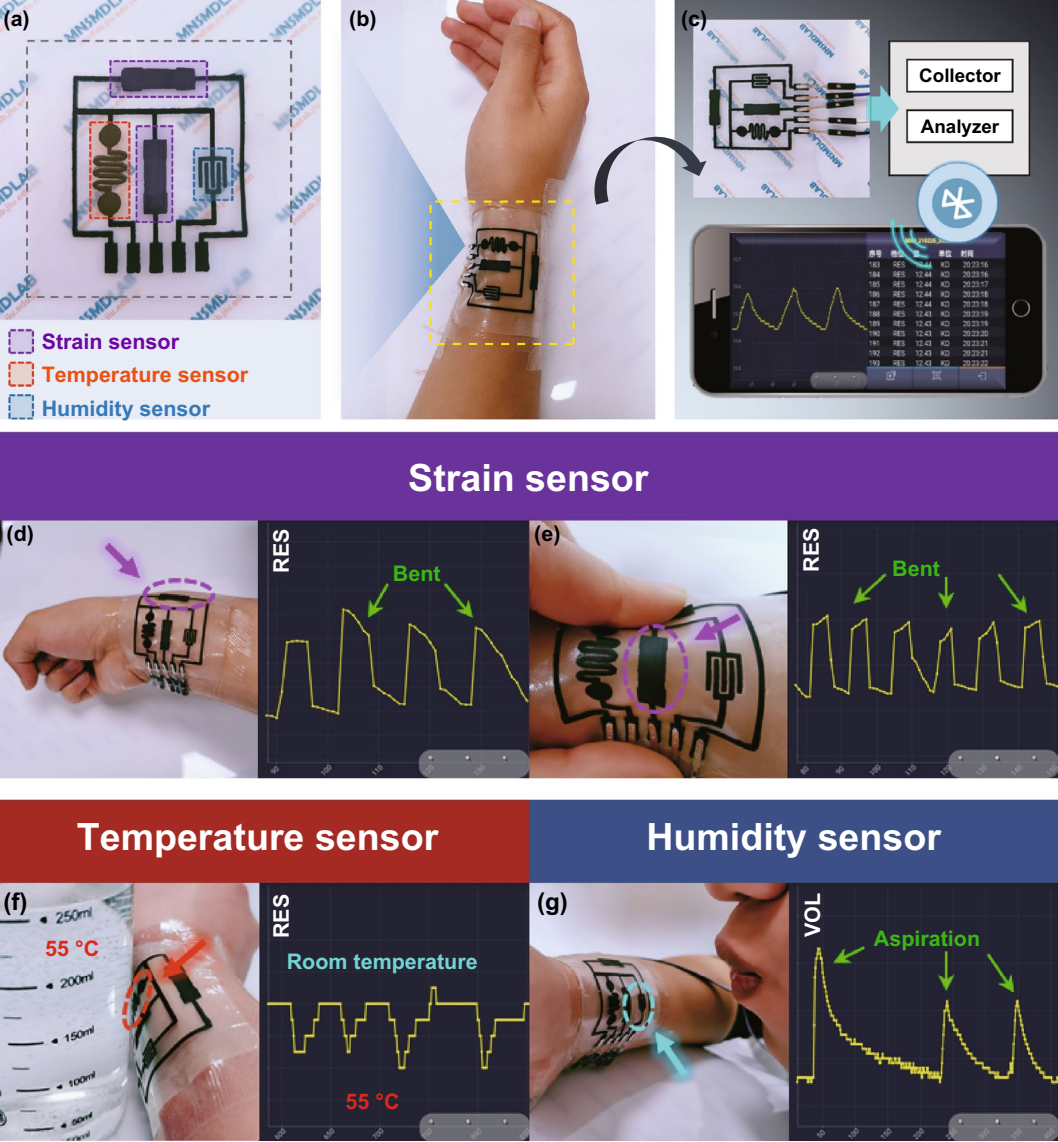
**a** Photograph of the e-skin containing strain sensors, temperature sensor and humidity sensor. **b** Photograph of the e-skin attached to the wrist. **c** Schematic illustration of wireless monitoring system of the e-skin. **d** Real-time response signals of wrist movement measured by the sensing unit and displayed on a phone. **e** Real-time response signals of skin deformation tested by the sensing unit and displayed on a phone. **f** Varieties in ambient temperature engendered by a cup with hot water near the temperature sensing unit and displayed on a phone. **g** Changes in ambient humidity caused by exhaling to the humidity sensing unit and displayed on a phone

To realize the monitoring of various external stimuli, strain, temperature and humidity sensors were integrated into one substrate to fabricate multifunctional e-skins. As shown in Fig. 6a, the e-skin comprised two perpendicular strain-sensitive units: a temperature-sensitive unit and a humidity-sensitive unit. All the functional units of the e-skin were screen-printed onto one CNF/PVA substrate with self-healing electrodes of the integrated circuit. Each part of the e-skin could be healed by spraying water. The e-skin was attached to the wrist and connected to signal acquisition and Bluetooth transmission equipment (Fig. 6b, c). Test curves can be directly displayed on a mobile device to realize real-time wireless monitoring. The above two strain sensors were used to monitor wrist movement (Fig. 6d) and skin deformation (Fig. 6e). The temperature sensor was employed to monitor changes in the ambient temperature. A cup containing water with a temperature of 55 ℃ was applied as the heating source, nearing and departing from the sensor, thus resulting in a temperature change near the sensor (Fig. 6f). The humidity sensor was used to detect ambient humidity changes, and exhaled air with a high humidity caused a drastic and rapid increase in humidity near the sensor (Fig. 6g). In the above multifunctional e-skin, any cross-interference between the different external stimuli was suppressed via reasonable material selection and structural design of each functional unit. The electrodes, temperature-sensitive unit and humidity-sensitive unit were insensitive to strain. Temperature almost does not affect the performance of the strain-sensitive unit. Interference of the RH with the electrodes and strain- and temperature-sensitive units could be avoided via encapsulation. Regarding the humidity-sensitive units on e-skin, the response value could be corrected when the strain and temperature are known.

## Conclusion

In summary, we developed a simple, clean and universally applicable method for the fabrication of e-skins with an excellent self-healing ability. CNF/PVA composite material was applied as both the substrate and binder in the functional layers. Self-healable electronics with various functional units were fabricated with the screen-printing technique. By adjusting the employed carbon materials and the electrode structure, various functional units (electrodes and strain, temperature and humidity sensors) were fabricated, and they exhibit an outstanding performance and self-healing ability. Both the functional layers and substrate could be self-healed in approximately 10 min by spraying water onto the damaged section. In particular, the various devices maintained their sensitivity and stability after self-healing even under large deformation. The strain sensor could achieve real-time monitoring of whole-body physiological signals via the detection of small skin deformation. In addition, a multifunctional self-healing e-skin was obtained through the integration of various functional units. The e-skin was combined with conventional electronics to transmit data to nearby smartphones to accomplish real-time wireless monitoring of various external stimuli. These results illustrate the potential application of our self-healing sensors and multifunctional e-skins in the fields of health monitoring and human–machine interaction. Although there are certain aspects of the e-skin that should be further optimized and improved, such as the sensing performance of temperature- and humidity-sensitive units, as well as comfortableness of wearable test systems, they can promote the research of e-skins to pay more attention to their practicality, such as wearability, biocompatibility, environmental friendliness and scalability, while pursuing a high sensing performance.

## Supplementary Information

Below is the link to the electronic supplementary material.

Supplementary file1 (MP4 15802 kb)

Supplementary file2 (PDF 969 kb)
