# Supplementary material for: Biocompatible Multifunctional E-Skins with Excellent Self-Healing Ability Enabled by Clean and Scalable Fabrication
Source: Nanomicro Lett. 2021 Sep 22;13:200. doi: 10.1007/s40820-021-00701-8 (PMC8458512; doi:10.1007/s40820-021-00701-8)
Supplement: Supplementary file 2 — Supplementary file2 (PDF 969 kb) [file 40820_2021_701_MOESM2_ESM.pdf]

Supporting Information for

Biocompatible Multifunctional E-Skins with Excellent Self-Healing Ability Enabled by Clean and Scalable Fabrication

Xiuzhu Lin[1], Fan Li[1], Yu Bing[1], Teng Fei[1], Sen Liu[1], Hongran Zhao[1,*], and Tong Zhang[1,*]

[1]State Key Laboratory of Integrated Optoelectronics, College of Electronic Science and Engineering, Jilin University, Changchun 130012, P. R. China

*Corresponding authors. E-mail: zhangtong@jlu.edu.cn (Tong Zhang), zhaohr@jlu.edu.cn (Hongran Zhao)

Supplementary Tables and Figures

Table S1 Performance parameters comparison of self-healing ability

Materials	Healing mechanism	Healing condition/time	Healing efficiency	Application	Refs.
MDPB/TDF	Diels-Alder reaction	Heating 105 °C/30 min	86%	Electrodes, strain sensor	[S1]
µNi/S	Hydrogen bond	50 °C/10 min	90%	Strain sensor	[S2]
PPy-DCh/PAA-Fe^{3+}	Ionic interactions	RT/2 min	96%	3D printing, strain sensor	[S3]
(bPEI/PAA-HA) ∗50	Hydrogen bond	Water/2 min	-	Electrodes	[S4]
sh-crl-PU	Hydrogen bond	RT/16 h	97%	Pressure/strain, temperature and VOCs' sensors	[S5]
Glycerol/HEC	Hydrogen bond	50% RH and 25 °C/8 min	96%	Electrode, electronic skin	[S6]
PDA@ENR/ CNT-Fe^{3+}	Metal coordination bond	RT/24 h	89%	Strain sensor	[S7]
PU/Graphene	Diels-Alder reaction	65 °C/5 h and microwave/ 5min	100%	Strain sensor	[S8]
Chi-Ph/ DF-PEG	Imine bond	Visible light/6 h	-	3D printing	[S9]
CNF/PVA	Hydrogen bond	Water/10 min	87%	Electrodes, multifunctional e-skin	This work

MDPB (monomers (1, 10- (methylene di-4, 1-phenylene) bismaleimide); TDF (2, 2'-(Thiodimethylene) difuran); μNi (micro-nickel); S (supramolecular organic polymer synthesis by diacids, triacids and diethylenetriamine); DCh (decorated chitosan); PAA (poly(acrylic acid)); bPEI (branched poly(ethylenimine)); HA (hyaluronic acid); sh-crl-PU (self-healing disulfide-cross-linked polyurethane); HEC (self-healing disulfi de-cross-linked polyurethane); PDA (polydopamine); ENR (epoxidized natural rubber); Chi-Ph (phenol-functionalized chitosan); DF-PEG (dibenzaldehydeterminated telechelic poly(ethylene glycol)).

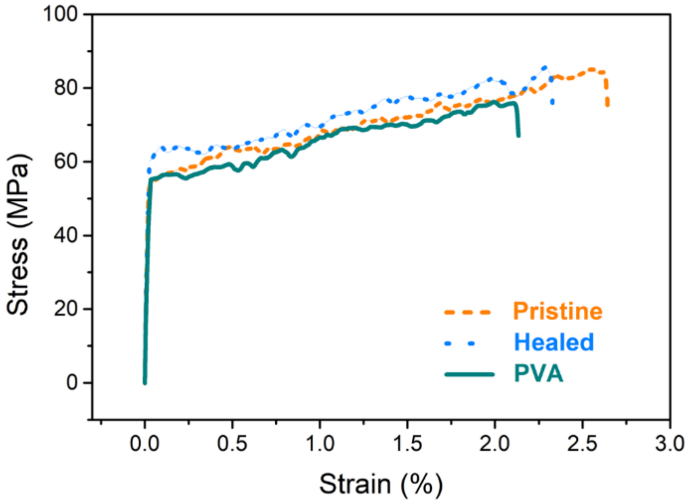

Fig. S1 Stress-strain curves of PVA film and pristine and healed PVA/CNF composite films

For PVA, the peak at 3350 cm^{-1} was attributed to O-H stretching, the characteristic peak of C-H wagging was observed at 1325 cm^{-1}, and the peak of stretching vibration of C−O was appeared at 1092 cm^{-1}. For CNFs, the characteristic peaks at 1060 and 1030 cm^{-1} represent the skeletal vibration of C-O-C pyranose ring. Since the content of CNF in the CNF/PVA composite film is little, and the position of characteristic peaks of CNF and PVA are close, the characteristic peaks of CNF in the composite film was drowned by the PVA, resulting in the peak position was not obvious.

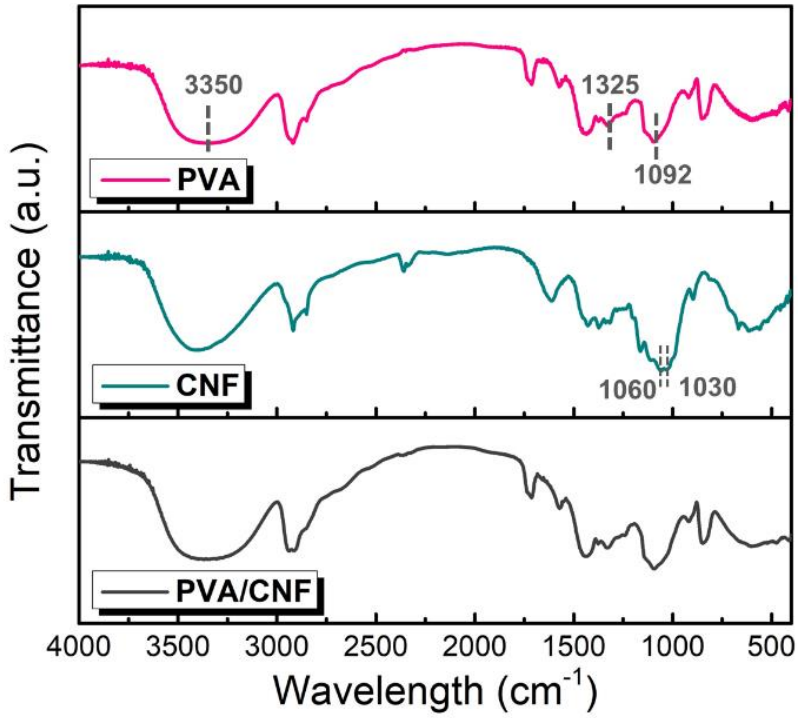

Fig. S2 FT-IR spectra of CNF, PVA, and the CNF/PVA composite

For the CNF/PVA composite film, the mass change in the temperature range from 50 to 150 °C is caused by water evaporation, the thermal decomposition temperature of the composite is about 260 °C.

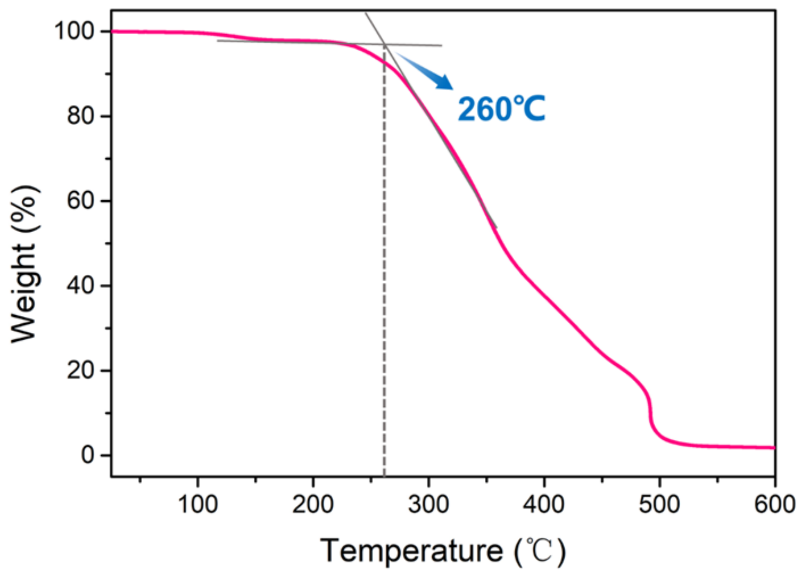

Fig. S3 TGA curve of the CNF/PVA composite film

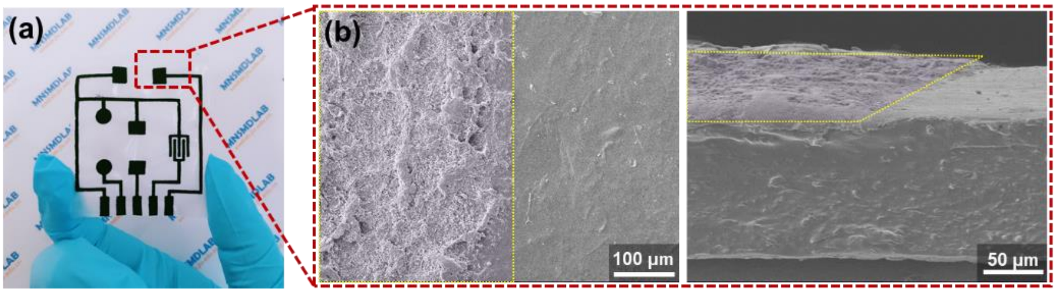

Fig. S4 a Optical image of electrodes screen-printed on CNF/PVA film. **b** Vertical (left) and cross-sectional (right) SEM images of electrodes on the CNF/PVA substrate

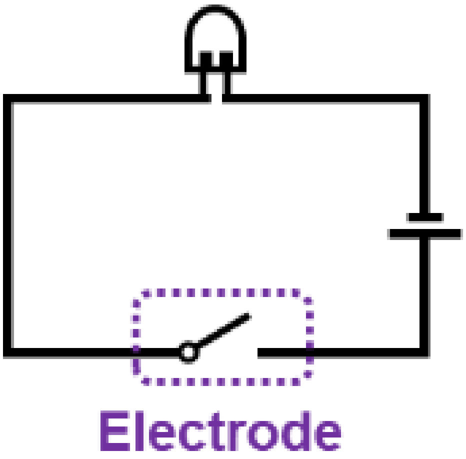

Fig. S5 Schematic diagram of equivalent circuit of Fig. 2a

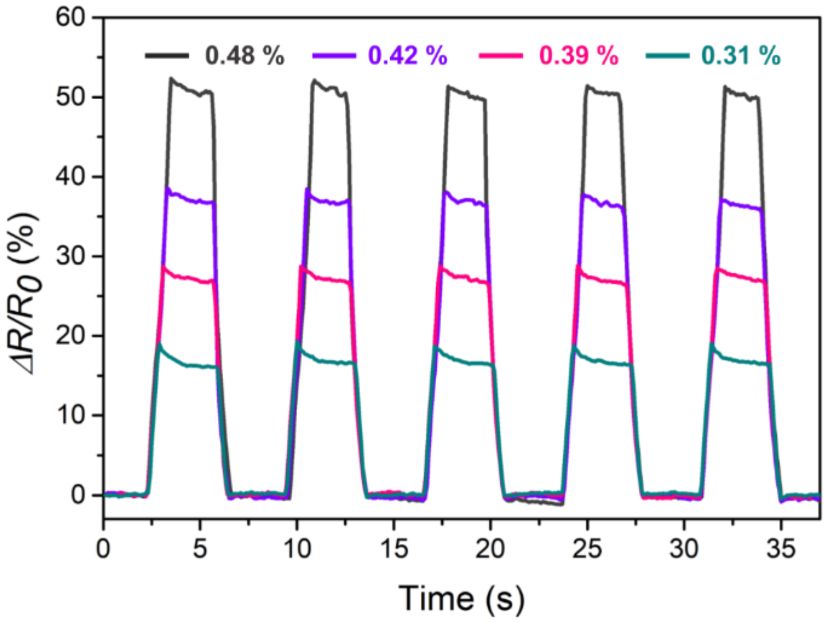

Fig. S6 Response curves of S-50 under strain of 0.31%, 0.39%, 0.42% and 0.48% for five cycles

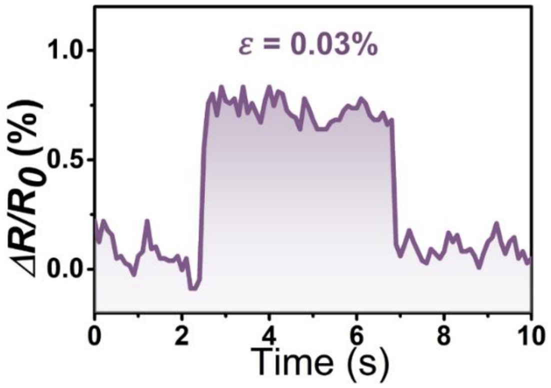

Fig. S7 The minimum detection limit of the S-50 sensor

The strain on the film is relative to the ratio of its thickness to its length. A CNF/PVA film with 3.75 mm in length and approximately 96.28 μm in thickness was applied to reach a much higher strain (2.64%) than that under general measurement conditions of strain sensors to investigate the effect of strain on the film thickness. Cross-sectional SEM images of flat and bending CNF/PVA films are shown in Fig. S9. The average thickness of the flat film is approximately 96.28 μm (Fig. S9a). In the SEM image of the bending film (Fig. S9b), five positions are selected to investigate the thickness distribution of the CNF/PVA film in the bending state. Based on the enlarged images, it is found that the maximum thickness variation occurs in the middle section, and the maximum thickness change caused by the applied strain is calculated to reach approximately 1.39%. According to Eq. (2), the calculated strain is proportional to the film thickness.

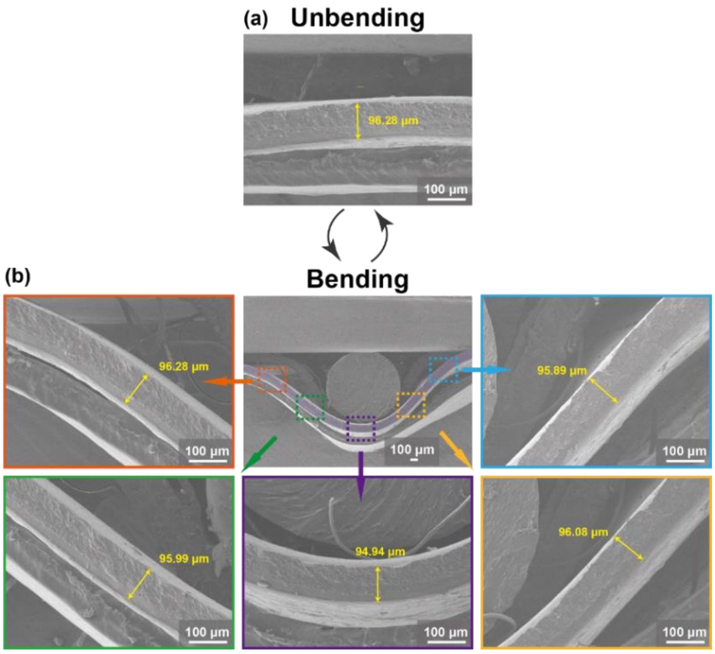

Fig. S8 a SEM image of cross section of CNF/PVA film in unbending state. **b** SEM images of cross section of CNF/PVA film in bending state and enlarged view at different positions

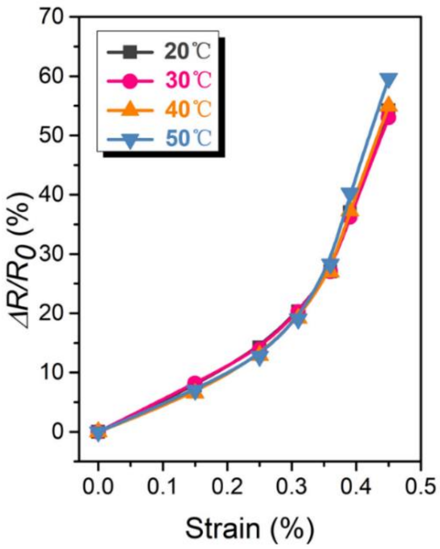

Fig. S9 Response-strain curves of S-50 sensor under various temperature

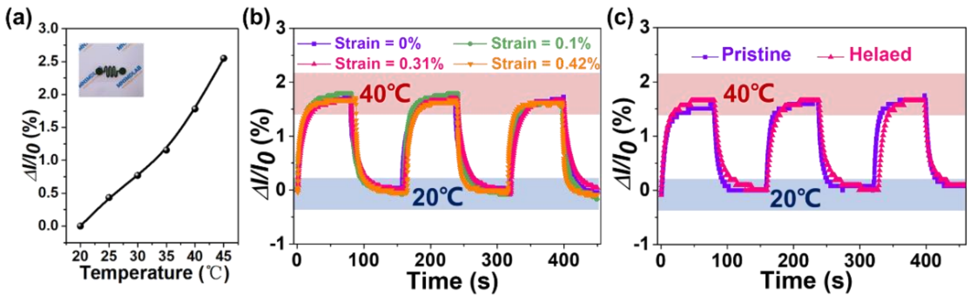

Fig. S10 a The response of the temperature sensor in the temperature range from 20 to 45 °C. **b** The dynamic response curves of the temperature sensor in three cycles during heating and cooling between 20 and 40 °C under different strains. **c** The real-time response curves of pristine and self-healed temperature sensor in three cycles during heating and cooling between 20 and 40 °C

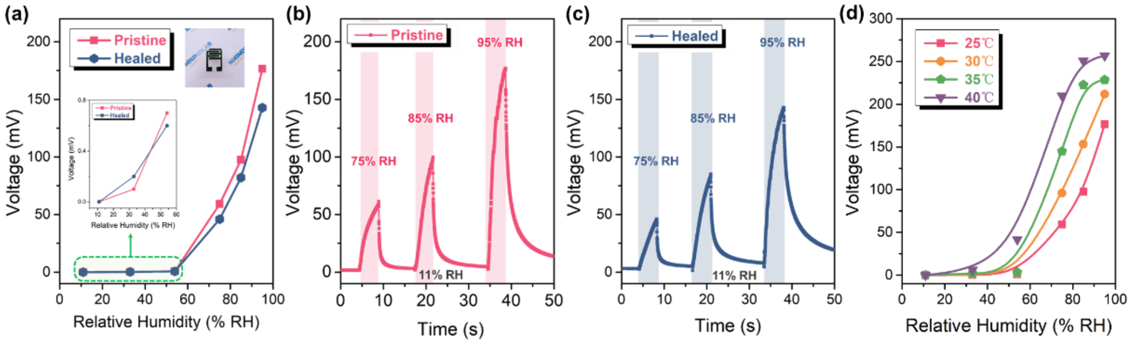

Fig. S11 a The response of the pristine and self-healed humidity sensor under the relative humidity from 11% RH to 95% RH. **b-c** The real-time voltage signals of pristine and healed sensors, respectively, at the relative humidity from 75% RH to 95% RH. **d** The response of the self-healed humidity sensor under the relative humidity from 11% RH to 95% RH in different temperature (from 25 to 40°C)

Supplementary References

[S1] Y. Yang, B.P. Zhu, D. Yin, J.H. Wei, Z.Y. Wang et al., Flexible self-healing nanocomposites for recoverable motion sensor. Nano Energy **17**, 1-9 (2015). http://doi.org/10.1016/j.nanoen.2015.07.023

[S2] B.C-K. Tee, C. Wang, R. Allen, Z.N. Bao, An electrically and mechanically self-healing composite with pressure- and flexion-sensitive properties for electronic skin applications. Nat. Nanotechnol. **11**, 825 (2012). https://doi.org/10.1038/NNANO. 2012.192

[S3] M.A. Darabi, A. Khosrozadeh, R. Mbeleck, Y.Q. Liu, Q. Chang et al., Skin-inspired multifunctional autonomic-intrinsic conductive self-healing hydrogels with pressure sensitivity, stretchability, and 3D Printability. Adv. Mater. **29**, 1700533 (2017). https://doi.org/10.1002/adma.201700533

[S4] Y. Li, S.S. Chen, M.C. Wu, J.Q. Sun, Polyelectrolyte multilayers impart healability to highly electrically conductive films. Adv. Mater. **24**, 4578-4582 (2012). https://doi.org/10.1002/adma.201201306

[S5] T.-P. Huynh, H. Haick, Self-healing, fully functional, and multiparametric flexible sensing platform. Adv. Mater. **28**, 138-143 (2016). https://doi.org/10.1002/adma. 201504104

[S6] M. M. Hao, L. H. Li, S. Q. Wang, F. Q. Sun, Y. Y. Bai et al., Stretchable, self-healing, transient macromolecular elastomeric gel for wearable electronics. Microsyst. Nanoeng. **5**, 9 (2019). https://doi.org/10.1038/s41378-019-0047-4

[S7] Y.Y. Han, X.D. Wu, X.X. Zhang, C.H. Lu, Self-healing, highly sensitive electronic sensors enabled by metal-ligand coordination and hierarchical structure design. ACS Appl. Mater. Interfaces **9**, 20106-20114 (2017). https://doi.org/10.1021/acsami.7b05204

[S8] J.H. Li, Q. Liu, D. Ho, S.F. Zhao, S.W. Wu et al., Three-dimensional graphene structure for healable flexible electronics based on Diels−Alder chemistry. ACS Appl. Mater. Interfaces **10**, 9727−9735 (2018). https://doi.org/10.1021/acsami.7b19649

[S9] Y. Liu, C.W. Wong, S.W. Chang, S.H. Hsu, An injectable, self-healing phenol-functionalized chitosan hydrogel with fast gelling property and visible light-crosslinking capability for 3D printing. Acta Biomater. **122**, 211–219 (2021). https://doi.org/10.1016/j.actbio.2020.12.051
